# Satellitome Analysis of the Pacific Oyster *Crassostrea gigas* Reveals New Pattern of Satellite DNA Organization, Highly Scattered across the Genome

**DOI:** 10.3390/ijms22136798

**Published:** 2021-06-24

**Authors:** Monika Tunjić-Cvitanić, Juan J. Pasantes, Daniel García-Souto, Tonči Cvitanić, Miroslav Plohl, Eva Šatović-Vukšić

**Affiliations:** 1 Division of Molecular Biology, Ruđer Bošković Institute, 10000 Zagreb, Croatia; mtunjic@irb.hr (M.T.-C.); plohl@irb.hr (M.P.); 2 Centro de Investigación Mariña, Universidade de Vigo, Dpto de Bioquímica, Xenética e Inmunoloxía, 36310 Vigo, Spain; pasantes@uvigo.es; 3 Genomes and Disease, Centre for Research in Molecular Medicine and Chronic Diseases (CIMUS), Universidade de Santiago de Compostela, 15706 Santiago de Compostela, Spain; danielgarciasouto@gmail.com; 4 Department of Zoology, Genetics and Physical Anthropology, Universidade de Santiago de Compostela, 15706 Santiago de Compostela, Spain; 5 Rimac Automobili d.o.o., Ljubljanska ulica 7, 10431 Sveta Nedelja, Croatia; tonci.cvitanic@gmail.com

**Keywords:** satellite DNA, satellitome, mobile element, Helitron, bivalve, *Crassostrea gigas*

## Abstract

Several features already qualified the invasive bivalve species *Crassostrea gigas* as a valuable non-standard model organism in genome research. *C. gigas* is characterized by the low contribution of satellite DNAs (satDNAs) vs. mobile elements and has an extremely low amount of heterochromatin, predominantly built of DNA transposons. In this work, we have identified 52 satDNAs composing the satellitome of *C. gigas* and constituting about 6.33% of the genome. Satellitome analysis reveals unusual, highly scattered organization of relatively short satDNA arrays across the whole genome. However, peculiar chromosomal distribution and densities are specific for each satDNA. The inspection of the organizational forms of the 11 most abundant satDNAs shows association with constitutive parts of Helitron mobile elements. Nine of the inspected satDNAs are dominantly found in mobile element-associated form, two mostly appear standalone, and only one is present exclusively as Helitron-associated sequence. The Helitron-related satDNAs appear in more chromosomes than other satDNAs, indicating that these mobile elements could be leading satDNA propagation in *C. gigas.* No significant accumulation of satDNAs on certain chromosomal positions was detected in *C. gigas*, thus establishing a novel pattern of satDNA organization on the genome level.

## 1. Introduction

Large fractions of eukaryotic genomes are composed of repetitive DNA sequences that could be either repeated in tandem, among which satellite DNAs (satDNAs) dominate, or are interspersed, due to the activity of mobile elements [1,2,3,4,5]. Nowadays, comprehensive analyses of repetitive DNA in eukaryotic genomes have been enabled by the employment of NGS (Next Generation Sequencing) methodologies, complemented with specialized bioinformatic tools and programs [6,7]. NGS-related bioinformatics allowed revealing either the complete set of repetitive DNA sequences, the repeatome [8], or the broad collection of satDNAs, the satellitome [9], present in eukaryotic genomes. These approaches showed surprisingly large numbers of satDNAs in eukaryotic genomes, i.e., 62 in the migratory locust *Locusta migratoria* [9], 129 in the Australian morabine grasshoppers of the genus *Vandiemenella* [10], 164 in the characiform fish *Megaleporinus microcephalus* [11] and 37 in the plant *Passiflora organensis* [12]. Bioinformatic approaches combined with fluorescence *in situ* hybridization (FISH) yielded new information about the structure, the chromosomal location, and the evolution of these sequences within or among genomes [13,14,15,16].

From the generally accepted point of view, classical satDNAs are organized in long arrays, consisting of hundreds to thousands of monomers repeated in tandem, and occupying the constitutive heterochromatin. Heterochromatin is mostly located at pericentromeric and subtelomeric chromosomal domains, being sometimes also found at interstitial loci of the chromosomal arms [4]. However, satDNA sequences were also detected outside of the heterochromatin, in different organizational forms. In some cases, the same sequence can simultaneously appear in the genome in the form of a classical satDNA, and in the form of short arrays, or as single monomers and monomer fragments located in euchromatic genome compartments [17,18,19,20,21]. Some short arrays are at the same time structural components of the central parts of mobile elements [17,22,23,24]. Due to this diversity, the study of the biology of satDNA sequences requires a versatile pool of model systems.

Bivalve mollusks hold great economic and ecological importance. Their commercial significance is unquestionable in aquaculture, where they have several million-ton productions per year due to their high nutritional value [25]. The ecological impact of these organisms is emphasized when invasive bivalve species start to occupy new environments, significantly affecting native organisms in the new habitat [26]. The research interest encompassing all aspects of bivalve biology is fast-growing [27,28], and is accompanied by an increasing number of sequenced genomes (31 until March 2021, NCBI) forwarding the bivalves rapidly towards well-established model organisms [29].

The estimated content of repetitive DNA in the majority of bivalve genomes sequenced so far is high, about 35%, while the contribution of the satDNA fraction is low, less than 2% of the genomic DNA (i.e., [30,31,32,33]). Although satDNA content is regularly underestimated in sequenced genomes, the results of classical restriction enzyme digestion and cloning are roughly in agreement with this estimation. The 26 different satDNAs from 48 bivalve species experimentally detected so far showed low genomic contents, some of them significantly less than 1% of the genomic DNA (reviewed in [34]). Altogether, this indicates that the presence of numerous, low copy satDNAs together with abundant mobile elements could be a specificity of bivalve genomes.

As the unambiguous classification of repetitive sequences in bivalve genomes is often difficult, many of them, about 70%, remained unassigned in reported cases [30]. For instance, although in *C. gigas*, the first sequenced bivalve genome, 36% of the assembled sequences were identified as repetitive, 62% of them could not be assigned to any of the known categories. Among those assigned, the majority were characterized as mobile elements, while satDNAs were estimated to make only 1.2% of the genome [35]. Nevertheless, tandem repeats belonging to the most abundant HindIII/Cg170 satDNA, were experimentally estimated to build 1–4% of the genome [36]. Short arrays of satDNA belonging to this sequence, in average about six monomers long, were regularly found in central parts of mobile elements belonging to the family of Helitrons/Helentrons [19,37], known to employ rolling circle mechanisms in their spreading process [38].

In accordance, the newly assembled *C. gigas* representative genome has a remarkably high number of predicted Helitron-related sequences when compared to several other molluscan genome assemblies [39]. Such a hybrid structure of mobile elements holding tandem repeats could explain the difficulties in both categorizing repetitive sequences in bivalves and determining the precise contribution of each type to the repeatome. For example, after the RepeatExplorer analysis, tandem repeats from central parts of sequences (later recognized as Helitrons) were placed into one cluster and classified as a satellite DNA, while sequences surrounding these central repeats were assigned to other, non-classified, clusters [35]. In that respect, only more detailed analysis of such sequences could resolve whether they appear in the form of the classical long-array satDNAs, short-array satDNAs, long-array satDNAs that are associated with mobile elements, short-array satDNAs associated with mobile elements, or in all of the abovementioned forms.

Despite extensive satellitome and repeatome studies in many species, little is known about how repetitive DNA sequences are structured in bivalves and thus need to be explored in detail at the whole-genome level. The aforementioned characteristics qualify *C. gigas* as a valuable non-standard model species in exploring both the satellitome and the organizational patterns of repetitive DNA sequences. In this study, the satellitome of a bivalve species, the Pacific oyster *C. gigas*, was analyzed, for the first time in our knowledge, using bioinformatic approaches accompanied by *in silico* and *in situ* chromosomal localization of the most prominent sequences repeated in tandem. Furthermore, in order to better understand the evolutionary processes structuring *C. gigas* genome, we disclosed the preferential organizational forms of the most abundant tandem repeats in this species.

## 2. Results

### 2.1. SatDNA Content of the C. gigas Genome

**Figure 1:**
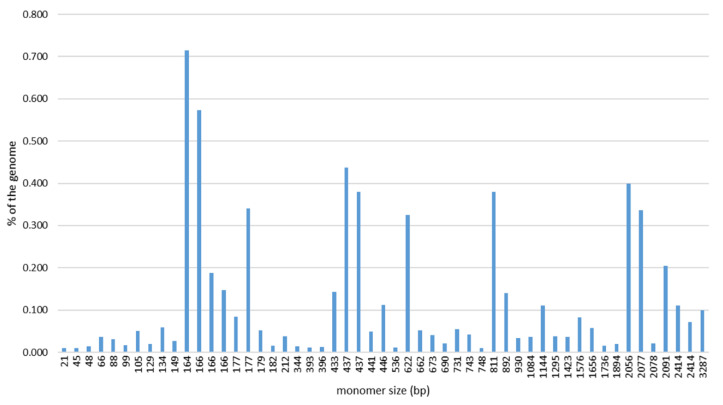
Monomer sizes (bp) and genomic contributions (%) of the 52 satDNAs of *C. gigas* including 2 variants of CgiSat01.

In order to obtain a comprehensive overview of the satDNAs in the Pacific oyster genome, several rounds of RepeatExplorer2 clustering were performed on four randomly subsampled sets of pair-end NGS reads. The combined results of the four analyses (Table S1) resulted in a pool of 52 sequences repeated in tandem that was considered as the satellitome of this species. The detected satDNAs exhibit a broad range of monomer lengths, varying from 21 (CgiSat43) to 3287 bp (CgiSat38), and AT contents (from 44 to 76.1%; Table 1). SatDNAs with 160–180 bp monomer lengths dominate (Figure 1), constituting 33% of the satellitome.

The abundance of satDNAs comprising the satellitome (averaged from the outputs of the four analyses) is relatively low, ranging from 0.01% (which is the bottom cut-off level of the program output) to 0.72% of the genome (Table 1).

CgiSat01a and CgiSat01b are variants (sub-families) of the Cg170/HindIII repeat family described by Clabby et al. [36] and López-Flores et al. [40] and confirmed as the most abundant tandem repeat of this species [35]. A few satDNAs correspond to several clusters of Helitron-incorporated tandem repeats reported by Vojvoda Zeljko et al. [37]; in particular, CgiSat01 corresponds to CL1, 2, 10 and 13, CgiSat08 and CgiSat37 to CL 3 and CL 7, respectively, and CgiSat09 to sequences from CL10 and 13. CgiSat17, CgiSat28 and CgiSat46 hold similarity to three clusters of sequences (Cl 112, 460, 150, respectively) enriched in the sample of the immunoprecipitated, CenH3-associated DNA sequences of the Pacific oyster [35].

The 52 satDNAs build 6.33% of the *C. gigas* genome. Consensus sequences of the satDNA monomers were used for screening of Repbase [41], a database holding different types of repetitive sequences and mobile elements. The search revealed that the satDNA sequences constituting 91.76% of the satellitome show similarity to sequences annotated as different mobile elements. Most of them, 75.61% of the satellitome, are showing similarity to the central repeats of Helitron mobile elements. For the rest of the mobile elements, the similarity was fragmentary and limited to only a segment of the satDNA monomer sequence.

### 2.2. In Silico Chromosomal Localization of C. gigas satDNAs

**Figure 2:**
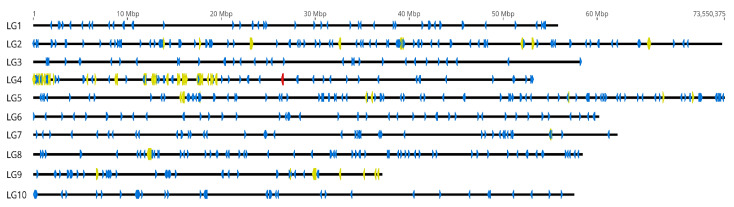
*In silico* localization exampled by three satDNAs annotated on the chromosomes of the *C. gigas* currently representative genome assembly GCA_902806645.1. CgiSat03 (blue), CgiSat27 (yellow) and CgiSat22 (red).

The distribution of the 52 satDNAs on the chromosomes of the Pacific oyster was inspected by an *in silico* analysis. For that purpose we annotated consensus sequences of each satDNA (allowing 70% similarity to detect different variants of monomer sequence) on the two currently available chromosome assemblies of *C. gigas*. The genome assembly holding acc. number GCA_902806645.1 [39] consists of ten chromosomes (linkage groups LG1-LG10) and 226 unplaced scaffolds, while the assembly under acc. number GCA_011032805.1 reports only ten chromosomes and no additional data. As shown in Figure 2, satDNAs display differences in chromosome placement, dissemination, and monomer grouping. For instance, CgiSat03 is highly interspersed throughout all chromosomes of *C. gigas*, CgiSat27 is interspersed on 6 chromosomes, while CgiSat22 is limited to a single locus on one chromosome. The *in silico* chromosomal localization of all satDNAs is presented in Figure S1.

Presence or absence of each of the 52 satDNAs on each chromosome were noted for the two genome assembly datasets together with the number of annotated monomers on chromosomes and unplaced scaffolds (Table 2). As CgiSat50 was not detected on any of the chromosomes of this species, an NCBI blast search was performed, revealing that this tandem repeat corresponds to the mitohondrial rDNA sequence. Twenty-five of the 52 satDNAs are widespread and appear on all chromosomes of the Pacific oyster, while the others are restricted to some or even a single chromosome.

The distribution patterns of the satDNAs were also inspected in regard to their connection to the mobile elements reported in Table 1. When the complete pool of mobile element-related satDNAs was checked, they exhibited no advantage in dispersal as the average number of the ocuppied chromosomes was 7.3 for mobile element-related satDNAs, and 7.0 for unrelated ones. However, when only Helitron-related ones were taken into consideration, the average number of ocuppied chromosomes was higher, 9.7 (Figure S2).

Additionally, the satDNAs present on a single chromosome in both assemblies, without hits in the unplaced scaffolds, provided the opportunity to associate some linkage group-based chromosomes of the GCA_902806645.1 dataset to the chromosomes of the GCA_011032805.1 assembly demonstrating that the profiles of CgiSat22, 26, 31, 41 and 52 are shared between LG4 and chr8, LG8 and chr4, LG1 and chr7, and LG2 and chr1 (Table 2).

### 2.3. Deciphering the Dominant Organizational Forms of C. gigas Tandem Repeats

Taking into consideration that a significant part of the *C. gigas* satellitome shows similarity to Helitron mobile elements (Table 1), we explored what the most common organizational form in which these sequences exist in this genome is: element-associated, standalone or both. Helitron elements usually consist of two well-structured left and right sequence segments (conserved boxes) and of a microsatellite followed by a short array of tandemly repeated satDNA monomers [17,37,38,42,43]. Table S2 displays the Helitrons showing similarity to *C. gigas* satDNAs after Repbase search. Sequence comparisons of those elements revealed high nucleotide sequence similarity in structural segments of some of them, primarily in the regions of the element ends, conserved boxes. Consequently, they can be divided into three groups. Helitron-N2_Cgi and N2C_Cgi share a 44 bp segment at the beginning of the elements (Box 1), a 49 bp segment at their ends (Box 2) and 156 bp between the microsatellite and the central repeats (Box 3). Helitron-N3_Cgi, N4_Cgi, N28_Cgi, N29_Cgi, N31_Cgi, N32_Cgi, N35_Cgi, N40_Cgi share the conserved 53 bp segment at the beginning (Box 4) and 42 bp at the end of the elements (Box 5). Nucleotide sequences of Boxes 1–5 are presented in Table S3. For Helitron-1 DEu, N25_Cgi, N62B_Cgi and N12_Cgi, the conserved boxes could not be determined, as they showed no similarity in terminal sequences among each other nor to the rest of the inspected Helitrons. Satellite DNAs showing similarity to the Helitrons whose conserved segments at the element ends could be determined were used for deciphering their most frequent organizational form. Eleven satDNAs meet this requirement: CgiSat01, 02, 03, 04, 05, 06, 07, 09, 14, 17 and 48, together constituting 64.25% of the satellitome. For that purpose, after the annotation of sequences of these satDNAs on chromosomes and scaffolds, the surrounding of each satDNA sequence (of at least one complete monomer), was inspected for the presence of conserved segments belonging to Helitron mobile elements. SatDNA sequences together with 2000 bp of left and right flanking regions were extracted from the currently representative chromosome assembly GCA_902806645.1 and searched for the presence of Boxes 1 & 2 or Boxes 4 & 5 that designate the element ends. The results are presented in Table S4. If the corresponding boxes were detected at each side of the repeat within the extraction, they were classified as element-associated. Structures having a box only on one side of the repeats were also included in this category, as truncation on one side of these elements is a rather frequent event [44]. If conserved boxes were not detected in the surrounding segments, repeats were classified as standalone.

**Figure 3:**
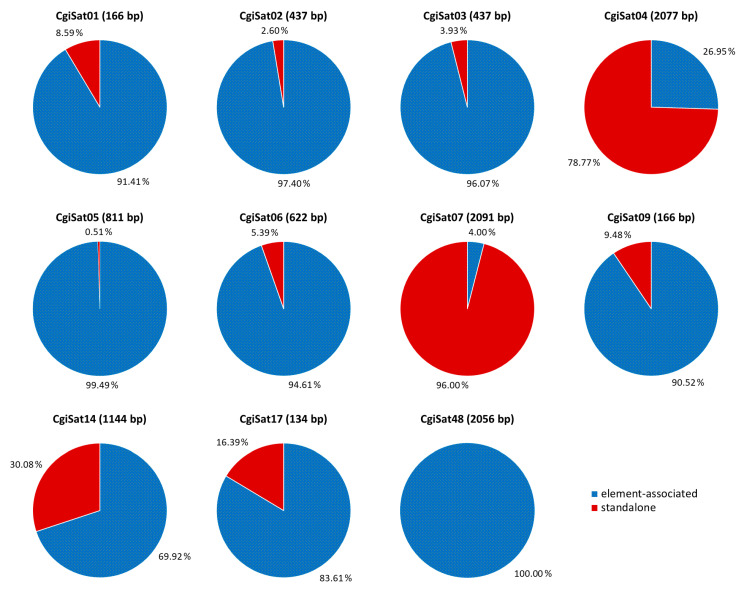
Proportions (%) of mobile element-associated (blue) and standalone (red) organizational forms in extractions corresponding to the satDNAs of *C. gigas*. Monomer sizes are provided in parentheses.

The main organizational forms of 11 satDNAs are depicted in Figure 3. Nine of them present mobile element association as dominant occurrence form, ranging from 69.9 (CgiSat14) to 100% (CgiSat48) of the extractions. Only CgiSat48 is exclusively mobile element-associated. Two satDNAs were dominantly found in standalone forms, CgiSat04 (78.77% of the extractions) and CgiSat07 (96.00%).

**Figure 4:**
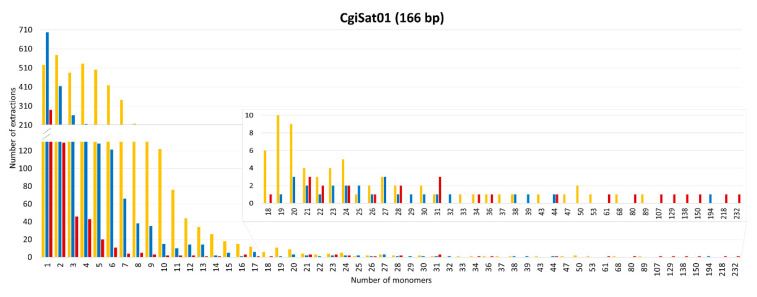

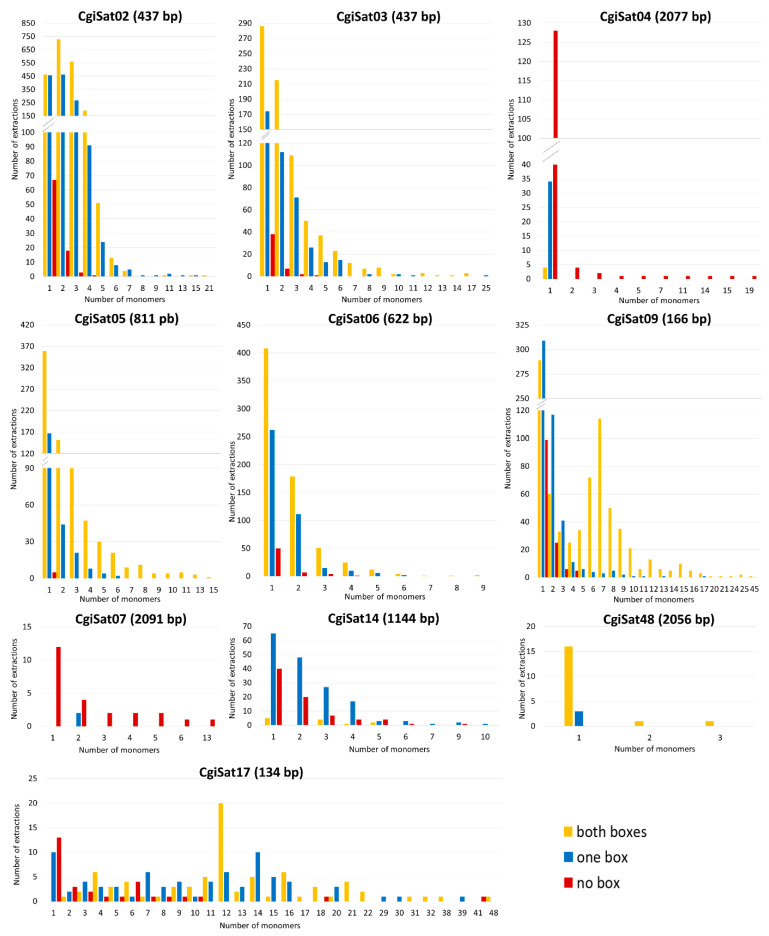
Number of satDNA monomers per extraction and number of extractions for the eleven closely inspected satDNAs of *C. gigas*. Presence or absence of Helitron boxes flanking satDNAs are presented by yellow (Helitron boxes at both sides), blue (Helitron boxes at one side) and red (no Helitron boxes) bars.

The number of monomers and the presence of the boxes for each satDNA were also analyzed for every extraction. Interestingly, single monomers, regardless of whether they are element-associated or standalone, were the most abundant category of extractions for the majority of the satDNA sequences, with the exception of CgiSat02 and CgiSat17 (Figure 4).

The most abundant satDNA of *C. gigas*, CgiSat01, presented the largest number of extractions and longest arrays of tandem repeats. For this satDNA the longest mobile element-associated array has 89 monomers while the longest standalone one is built of 232 monomers (about 40 kb). The latter array was found to hold an assembly gap, thus potentially forming an even longer stretch in the genome on LG3. Although characterized as a satDNA in the RepeatExplorer2 TAREAN analysis, arrays with more than three tandemly arranged repeats were not detected for CgiSat48. Its dominant form are single monomers with Helitron boxes present at both ends. It should be noted that CgiSat48 monomer repeats are unusually long, about 2 056 bp, and composed of the unique sequence that could not be resolved into potential subunits. For the rest of the inspected satDNAs, the maximum number of monomers in an array ranged from nine (CgiSat06) to 48 (CgiSat17). Maximum array length and mobile element-association do not seem to be interdependent. Namely, for all but CgiSat01 inspected satDNAs the longest array belongs to the dominant organizational form of the corresponding satDNA, regardless if it is mobile element-associated or standalone (Figure 4). It was also observed that the satDNA monomer size and the maximum number of monomers that can be found in an array are not interdependent (Figure S3).

### 2.4. Fluorescence In Situ Hybridization

**Figure 5:**
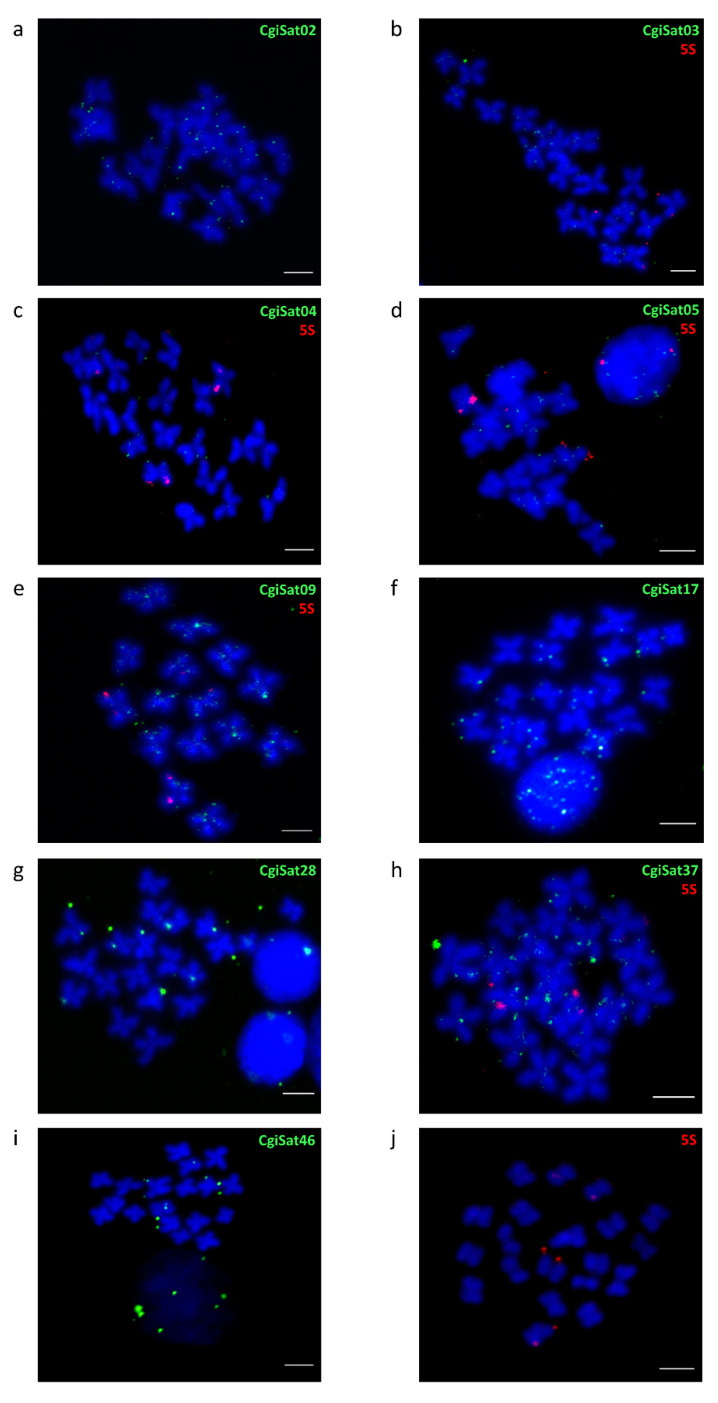
FISH localization of satDNA repeats belonging to (**a**) CgiSat02 (**b**) CgiSat03, (**c**) CgiSat04, (**d**) CgiSat05, (**e**) CgiSat09, (**f**) CgiSat17, (**g**) CgiSat28, (**h**) CgiSat37 (**i**) CgiSat46. To confirm the specificity of the interspersed signal 5S rDNA (**j**) was used as a positive control and co-hybridized with several satDNAs (**b**–**e**,**h**). Scale bars represent 3 µm.

The chromosomal distribution of the most abundant satDNA, CgiSat01, was already described by Wang et al. [45] and Tunjić Cvitanić et al. [35]. It displayed strong, discrete FISH signals in the centromeric regions of several chromosomes of *C. gigas*, together with highly interspersed signals on chromosome arms. Here, we performed fluorescence *in situ* hybridization on metaphase chromosomes for several of the most prominent satDNAs of the Pacific oyster (Figure 5a–i).

CgiSat02 and CgiSat09 present a substantial number of signals along chromosome arms. CgiSat03, 04, 05, 17 and 37 exhibit similar interspersed pattern but with reduced number of signals, which is even more limited for CgiSat28 and CgiSat46. To confirm the specificity of such signal distribution, a few satDNA probes were hybridized together with 5S rDNA probes as a control (Figure 5b–e,h). 5S rDNA is known to be present on chromosomes 4 and 5, exhibiting strong subtelomeric signals on one chromosome pair and weak ones on another [46], and the same distribution pattern is also noticeable in Figure 5j.

## 3. Discussion

In this work we present the satellitome of the Pacific oyster *C. gigas*, analyse the links between satDNAs and mobile elements, and define some general organizational features of the most prominent repetitive DNAs in this genome. Comprehensive satellitome studies performed recently on diverse species by using advanced methodological approaches highlighted not only the extraordinary diversity in composition and content of satDNAs within and among species but also indicated the sharp contrasts in their genomic arrangements [4,47].

Several characteristics qualify *C. gigas* as a valuable non-standard model species in exploring satellitome and repeatome organizational patterns: the low abundance of satDNAs vs. mobile elements [31,39], the low amount of heterochromatin, limited to the centromeric region of one chromosome pair and the telomeric region of another [35,48], the incorporation of short satDNA arrays into mobile elements of the Helitron/Helentron family [22,37], and the remarkably high number of predicted Helitron-related sequences [39]. *C. gigas* is also the first bivalve species in which the DNA composition of the centromeric regions and the heterochromatin was explored by using chromatin immunoprecipitation, revealing a predominance of DNA transposons and the lack of centromere-specific repetitive sequences [35].

The introduction of third generation sequencing methods, supplemented with novel mapping and bioinformatic tools, enabled reading long segments composed of satDNAs and filling the unassembled gaps left in earlier genome outputs, populated mostly by sequences repeated in tandem [49,50]. The sequencing and assembly of *C. gigas* genome is particularly demanding because of the high level of heterozygosity and the abundance of repetitive sequences. In the first release, those hindrances were solved by a combination of NGS, fosmid pooling, and hierarchical assembly [31,51]. More recently, the *de novo* sequencing and assembly of the *C. gigas* genome, employing a combination of high coverage long and short read data and linkage maps, resulted in a less fragmented genome and gained two assemblies at the chromosome level, GCA_902806645.1 [39] currently being the representative one.

In this work, the repetitive DNA content of *C. gigas* was accessed by low-coverage NGS followed by RepeatExplorer2 clustering. The detection of 52 satDNAs constituting the satellitome of the Pacific oyster (Table 1) is a giant leap in the knowledge about the satDNA content of this species, up until now limited to only three satDNAs (reviewed in [34]). The employment of NGS methods has immensely improved satDNA detection substantially increasing the number of satDNAs detected in the genomes of many species (e.g., [9,10,11,52,53]). Although satDNA monomer lengths vary significantly in the satellitome of the Pacific oyster, 160–180 bp-long monomers predominate. This monomer size, reflecting nucleosomal periodicity, is generally considered to be evolutionarily favored [54]. The same range of monomer sizes was also observed for a group of short satDNA arrays of *C. gigas* tandem repeats incorporated in Helitron/Helentron mobile elements [37]. Close connections between satDNAs and mobile elements have been observed in many forms and in many organisms (reviewed in [55]), including bivalves (reviewed in [34]). However, our work evidences an additional level of how vast and intimate the relation between tandem and interspersed repeats can be, as a large part of the *C. gigas* satellitome exhibits similarity to different mobile elements, especially to those of the Helitron type (Table 1).

As mobile elements were proposed to generate complex rearrangements and even facilitate genomic dispersal of satellite repeats [17,19,43,56,57,58], it could reasonably be expected that satDNAs connected to mobile elements would have some propagation and dissemination advantages. In *C. gigas*, only Helitron mobile element-related satDNAs populated higher number of chromosomes when compared to other satDNAs (Figure S2). The rest of the mobile element-related satDNAs present similarity to mobile elements only in parts of their monomer sequence, not being their constitutive part, thus making active propagation of these satDNAs via mobile elements unlikely. Monomers with such fragmentary similarities could be the result of the tandemization of a segment of the mobile element and a nearby sequence, the imprecise excision of mobile elements leaving behind some sequence segments, or some other sequence rearrangements. On the other side, the substantial contribution of the Helitron-related satDNAs to the satellitome of the Pacific oyster (75.61%) speaks in favor of these mobile elements being the main players in satDNA propagation in this organism.

However, the final number of chromosomes occupied by the mobile element-related and unrelated satDNAs has to be taken with reservations, as unplaced scaffolds still exist for the currently representative genome assembly, potentially broadening the span of chromosomes occupied by one or both groups of satDNAs.

After determination of the conserved boxes located at the ends of several Helitron elements related to 11 satDNAs, we were able to inspect what the dominant organizational form of those satDNA sequences are: element-associated, standalone, or present in both organizational forms. Interestingly, only CgiSat48 satDNA shows exclusively one organizational form, always being associated with a Helitron (Figure 3). One organizational form prevails in the remaining satDNAs, mobile element-association in eight out of eleven and standalone forms in two. The same sequence can obviously (co)exist in different organizational forms throughout the genome. Such lack of uniformity, and the parallel existence of several different organizational patterns presented by the 11 satDNAs, would suggest that the present complex organization of *C. gigas* tandem repeats is not a result of a single mechanism.

Such unusual organization of satDNAs, largely presenting tandem repeats within mobile elements and only a fraction existing as standalone arrays (Figure 3), significantly differ from the “classical” satDNA organization in long arrays, reported within a wide spectrum of organisms throughout the animal and plant kingdom (humans, insects, and plants; reviewed in [4]). In the special organizational form described in *C. gigas* only indications of classical satDNAs exist, while most of the tandem repeats are scattered throughout the genome (Figure S1 and Figure 5) without any significant grouping that would clearly distinguish heterochromatic and euchromatic genome compartments. This fact is complementary to the scarceness of heterochromatin in the Pacific oyster, limited to two small, (peri)centromeric and telomeric, segments on two chromosome pairs [35,48].

In regard to the mechanisms leading to such a distribution, several models for forming satDNA arrays from repeats present within mobile elements have been proposed. According to the model proposed by Hikosaka and Kawahara [59] for the satDNA formation from a Miniature Inverted-repeat Transposable Element (MITE), the occurrence of tandem repeats within the MITE element involves the formation of a stem-loop structure between two adjacent MITE elements on the single-stranded DNA, due to the delay of the DNA replication on one strand. This loop is cut out by a nuclease and the remaining strands are rejoined. As a consequence, the new MITE contains repeats from both previous elements. Further extension of the sequence could be accomplished by the same process. Although such sequences are still interspersed repeats, recombination processes could subsequently happen, and the sequences develop into longer arrays of tandem repeats and ultimately into satDNAs. Izsvak et al. [60] also proposed a mechanism based on a stem-loop structure to explain the formation of tandem repeats from a mobile element. During the replication of the MITE element, inverted repeats or palindromic sequences allow forming a stem-loop in the newly synthetized strand while still in the process of synthesis. Then, the whole structure is twisted back, and DNA synthesis continues at the 3′ end of the stem-loop, using the nascent strand as a new template. The duplicated segment is released in the form of an extrachromosomal stem-loop that is incorporated into a new site in the genome, facilitated by the local homology between the motifs in the target sequence and in the amplified extrachromosomal sequence. Structures in MITEs that enable formation of such stem-loop structures, like terminal inverted repeats, are also found at the end of Helitron elements and their structural variants, Helentrons [38].

Furthermore, Helitron/Helentron elements show additional mechanisms involved in their propagation and the amplification of the sequences within. They are known to capture segments of the host genome [38], frequently tandem repeats ([22,42,43,61,62,63], etc.). Helitrons transpose using a rolling circle replication (RCR) mechanism that initiates at the 5′-end and progresses towards the 3′-end, where the 3′ terminal hairpin structure serves as a recognition site for termination and cleavage [44]. Alternatively, during the replication, the original 3′ terminator can be deleted from the circular DNA template by an intramolecular recombination event between internally repeated 5′-ends. The next round of replication generates a tandem array of truncated Helitrons lacking 3′-ends. In the last step, the amplified single complete Helitron copy or multiple truncated Helitron copies are integrated into new genomic locations [44]. Such mechanism could also explain a large number of extractions found in our study, where satDNA sequences were found to be associated with Helitron box only on one side of an array/monomer (Figure 4). Alternatively, such arrays associated to only one box could also result from recombination events between element-associated and standalone arrays, generating hybrid structures. Further prolongation or shortening of the arrays, both element-associated and standalone, could happen via the usual mechanisms of unequal crossing-over exchanges, known to govern satDNA evolution, including repeated rounds of rolling circle replication and reinsertion (reviewed in [3]).

It is also possible that mobile element-associated and standalone forms of satDNA sequences of the Pacific oyster are interchangeable through the interplay of all previously mentioned mechanisms. Related to this, Scalvenzi and Pollet [57] explained two possible directions in the life of the tandem repeats. They propose that precursor satDNA sequences can be captured by a mobile element, followed by the amplification of tandem repeats within. Transposition of elements containing tandem repeats continues but, as the number of repeats within the element increases, the transposition rate of the element decreases. At the same time, recombination rates start to increase with the growing number of monomers, thus causing further expansions of the tandem repeats. Finally, mobile element-associated tandem repeats can give rise to the classical satellite DNA arrays, devoid of surrounding sequences by accumulating mutations over time.

CgiSat01 (corresponding to HindIII/Cg170 satDNA) could be an example for such a scenario. Mobile elements with one to ten internal repeats of CgiSat01 satDNA are present in a large number of copies in the genome due to their continuous transposition, which seems to decay as the number of internal repeats increases. Finally, arrays with more than 100 monomers are dominantly present in a standalone form (Figure 4).

Interestingly, single monomers were the most common extraction for 11 satDNAs inspected, with each satDNA having an individual ratio among extractions surrounded with 2, 1 or 0 mobile element-derived boxes (Figure 4). Such sequences represent the starting- and the end-point of the Scalvenzi and Pollet perpetual model [57], which could potentially be the reason for the large number of extractions holding monomers. Observed structures could have several potential origins. Monomers surrounded with 2 boxes could be a starting structure with possibility of array expansion, or generated after array reductions, while monomers surrounded by only one box or by no boxes could be a result of recombination events, excision events, array reductions, or box deterioration.

Recent studies of satDNA array organization from long-read sequencing data also presented different organization patterns in other organisms. In the plant *Lathyrus sativus*, 11 major satDNAs showed interesting differences between the analyzed repeats [24]. There, only two satDNAs were predominantly organized in long arrays typical for satDNA, while the remaining nine satDNAs were found to be derived from short tandem arrays located within LTR-retrotransposons, occasionally expanding in length. Likewise, in the *C. gigas* satellitome, if array length is taken into consideration, only one satDNA (CgiSat01) would be a candidate for a classical satDNA. However, 91.4% of the extractions holding this sequence were found to be mobile element-associated (Figure 3). On the other hand, two other satDNAs, CgiSat04 and CgiSat07, are dominantly in standalone form, yet their array lengths do not exceed 19 and 13 monomers, respectively, and their monomer sizes exceed 2000 bp. Although the most abundant satDNAs were studied in our work, the presence of classical satDNAs in the unstudied parts of the satellitome is still possible. However, in that case, the contribution of such sequences to the genome would be very limited, as the abundance of those satDNAs is very low. On the other hand, the detailed examination of the sequences constituting the centromeric and the heterochromatic genome components performed after chromatin immunoprecipitation with anti-CenH3 and anti-H3K9me3 antibodies in *C. gigas* is in line with the above discussed. Sequences building the centromeres were found to be quite heterogeneous and presented high dispersal throughout the genome, while the heterochromatin exhibited general paucity and was predominantly constituted of DNA transposons [35].

## 4. Materials and Methods

### 4.1. Sequencing and Read Clustering

Genomic DNA was extracted from adductor muscle tissue using the DNeasy Blood and Tissue Kit (Qiagen, Hilden, Germany) according to the protocol provided by the manufacturer. As oysters display high levels of phenotypic plasticity, DNA barcoding was performed for molecular identification and species confirmation. For that purpose, primers for the mitochondrial cytochrome c oxidase subunit 1 (COI) gene were used, LCO-1490 5′-GGT CAA CAA ATC ATA AAG ATA TTG G-3′ and HCO-2198 5′-TAA ACT TCA GGG TGA CCA AAA AAT CA-3′. PCR amplification was performed with an initial denaturation at 94 °C for 5 min, 35 cycles of 94 °C for 30 s, 52 °C for 30 s, 72 °C for 30 s, with a final extension at 72 °C for 10 min. PCR products were sequenced and compared with publicly available COI sequences of *C. gigas* from NCBI GenBank. Library preparation and Next-generation Illumina sequencing of C. gigas genomic DNA was performed on a HiSeqX platform by Admera Health facility (South Pleinfield, NJ, USA). Low-coverage sequencing was implemented, as significantly reduced genome coverage has been recommended for repetitive DNA analysis, due to their enrichment in respect to single-copy ones [64]. *C. gigas* genome was sequenced to about 1.5× coverage, generating 2 × 2,768,912 paired-end reads, 151 bp in length. Raw sequence reads can be found in NCBI under the BioSample accession number: SAMN15184427, BioProject: PRJNA638244.

Genomic repeat identification was performed using the RepeatExplorer2 pipeline [65] on the Galaxy server (https://repeatexplorer-elixir.cerit-sc.cz/galaxy/, accessed on 1 September 2020). For that purpose, genomic reads were quality-filtered, trimmed, interlaced and paired-end reads with no overlap were further processed. Similarity-based read clustering was performed under the default parameters, using several randomly subsampled sets: two of one million reads, one of two million reads and one set of 1,779,522 reads, corresponding to genome coverages of 0.2×, 0.4× and 0.35×, respectively.

### 4.2. Satellite DNA Analysis

TAREAN [64] incorporated into RepeatExplorer2 pipeline provided the consensus sequences of satDNA monomers. Consensus sequences of satDNAs obtained by four rounds of read clustering were compared to each other using discontinuous megablast with the default parameters in Geneious prime v. 2019.0.4 (Biomatters Ltd., Auckland, New Zealand) in order to detect clusters belonging to the same satDNA in different analyses. The same program was used for all subsequent sequence analysis and editing. Consensus sequences of the 52 satDNAs constituting the satellitome of *C. gigas* are available as Data S1. For the annotation of the 52 satDNAs, two publicly available chromosome-level assemblies of the Pacific oyster genome were downloaded from NCBI, GenBank assembly accession: GCA_902806645.1 [39] and GCA_011032805.1. Consensuses of monomer sequences were used for annotation of each satDNA on chromosomes and scaffolds, allowing 70% divergence to the consensus in order to encompass different sequence variants.

### 4.3. Analysis of the Flanking Regions of the satDNA Arrays

For the analysis of the flanking regions of the satDNA arrays and single monomers, the currently representative genome assembly GCA_902806645.1 [39] was used. It also contains the unplaced scaffolds, genome segments that are usually left unassembled, as they are particularly enriched in sequences repeated in tandem. The .csv file holding a list of exact positions of each annotated monomer for all identified satDNAs was exported from Geneious program. Left and right flanking regions, 2000 bp in length each, were excised along with the surrounded satellite DNA array or single monomer. For excision, we used a custom-made Python script (file parser.py). Input files for Python script were a .csv file containing the chromosome sequence and the file exported from Geneious, with the exact position of the monomers on each chromosome. The Python script output file was a fasta file of the extractions and a .csv file with the information regarding the position of a satellite sequence and the position of the 2000 bp of its left and right flanking regions. The excision holding flanking regions and satDNA array/single monomer was then used for annotation of boxes of interest using Geneious Prime software.

To generate a summary list of the boxes present in the flanking regions of each satDNA array/single monomer, another custom-made Python script was made (file boxer.py). The input file for this Python script was again a Geneious-generated .csv file, holding the information regarding the position of the annotated boxes in the flanking regions. In the Python script output table, the presence of a certain box within the flanking region of the satDNA array/single monomer was marked with 1, and the absence of a box was marked with 0. The same principle was applied for the excisions of satellite arrays and belonging flanking regions localized in scaffolds and for the detection of the boxes in the scaffold extractions (file parser_scaffolds.py, and file boxer_scaffolds.py). The extractions and annotations were additionally checked by eye. All scripts used are available at: https://bitbucket.org/MonikaTC/tunjic-cvitanic-et-al.-2021/src/master/ (uploaded 1 May 2021).

### 4.4. Mitotic Chromosomes Preparations

Juvenile specimens of the Pacific oyster were collected in Ria de Aveiro, Portugal. Laboratory tanks at 18 ± 1 °C with aerated and filtered seawater were used to feed the oysters with microalgae for seven days, in order to promote their growth and maturation. The mitotic chromosome preparations were obtained according to the protocol described in Martinez-Exposito et al. [66], with few modifications. Gills were excised, after a 12 h treatment of the specimens in a 0.005% colchicine solution. Hypotonic shock in 50% and 25% seawater (25 min each) was performed on gill tissue, followed by fixation in ethanol:acetic acid (3:1) for 1 h. Cell suspensions, obtained by exposing dissected gills to 60% acetic acid, were dropped onto slides preheated to 56 °C.

### 4.5. Probe Labelling

Probes for fluorescence *in situ* hybridization corresponding to CgiSat02, 03, 04, 05, 09, 17, 28, 37, 46 and 5S rDNA were labeled by PCR. The reactions contained 50 ng of DNA, 100 µM dATP, dGTP and dCTP, 65 µM dTTP, 2.5 mM MgCl2, 2.5 U GoTaq G2 Flexi Taq DNA polymerase, 1× GoTaq Flexi Reaction Buffer (all Promega, Madison, WI, USA), primers (1 µM each) and 35 µM biotin-16-dUTP (Jena Bioscience, Jena, Germany) for satDNAs or 35 µM digoxigenin-16-dUTP (Roche, Basel, Switzerland) for 5S rDNA, in 50 µL volumes. Nucleotide sequences of each primer pair used and PCR amplification conditions employed are presented in Table S5. Probe purification was performed using the QIAquick PCR Purification Kit (Qiagen, Hilden, Germany), following the protocol within. Probes were checked on 1% agarose gel and the concentration of the purified probes was measured using a Qubit Fluorometer. 30 ng of probe was used per FISH experiment.

### 4.6. Fluorescence In Situ Hybridization

Experiments were performed according to the protocol described in Pérez-García et al. [67], with the alteration in pepsin digestion (5 min at 37 °C). Prior to usage, DNA probes were denatured at 80 °C for 8 min and placed on ice for 2 min. Fluorescein-labelled avidin D and biotinylated anti-avidin D (both Vector Laboratories, Burlingame, CA, USA) were used in the signal detection process for biotin-labelled probes and anti-digoxigenin-rhodamine Fab fragments (Roche, Basel, Switzerland) for the digoxigenin-labelled probe. Counterstaining of chromosomes was performed using 100 ng/mL 4’, 6-diamidino-2-phenylindole (DAPI) (Sigma-Aldrich, St. Louis, MO, USA), and slides were subsequently mounted in Mowiol 4-88 antifade mounting medium (Sigma-Aldrich, St. Louis, MO, USA). For slide visualization and image capturing Nikon Eclipse-800 fluorescence microscope and a Leica TCS SP8 X laser-scanning microscope were employed.

## 5. Conclusions

The many peculiarities in genome organization already known for *C. gigas* were furthered in our satellitome analysis. We combined RepeatExplorer2 analysis with the assignment of the obtained sequences on two recent chromosome-level assemblies of *C. gigas*, followed by FISH localization of the most prominent satDNAs. The satellitome of *C. gigas* is composed of 52 sequences repeated in tandem that altogether build about 6.33% of the genomic DNA. SatDNAs are distributed along whole chromosomes presenting unusual interspersed patterns, with density and chromosomal distribution specific for each satDNA. In contrast with the established concept of satDNA genomic organization, no significant accumulation of satDNAs was observed in any preferred chromosomal position. Most arrays are relatively short and can be found either as standalone arrays or associated with conserved boxes characteristic for Helitron mobile elements that flank the arrays from one or both sides. Most of the inspected satDNAs are dominantly found in mobile element-associated form, but two of them mostly appear in a standalone form. Only one of the inspected repeats is present exclusively as element-associated. An advantage in the number of chromosomes occupied was observed for Helitron element-related satDNAs, speaking in favor of satDNAs in *C. gigas* being propagated with the aid of this family of mobile elements. No evident link between monomer length and the maximum number of monomers that can be found in an array was observed, and the longest array usually belongs to the dominant organizational form of that satDNA, regardless if it is element-associated or standalone. The lack of classical satDNAs in the pool of inspected satDNAs, the lack of uniformity in the organization, and the parallel existence of different organizational patterns within the satellitome, establishes *C. gigas* as a model organism of interest for further detailed studies of repetitive DNA biology.

## Figures and Tables

**Table 1:** Main characteristics of the 52 satDNAs constituting the satellitome of *C. gigas*.

satDNA	Monomer Length (bp)	% of the Genome (Average)	% of the Satellitome	% AT	Repbase
CgiSat01a	164	0.72	11.29	59.8	DNA/Helitron
CgiSat01b	166	0.57	9.04	57.2	DNA/Helitron
CgiSat02	437	0.44	6.91	65.7	DNA/Helitron
CgiSat03	437	0.38	6.00	67.5	DNA/Helitron
CgiSat04	2077	0.34	5.32	67.3	DNA/Helitron
CgiSat05	811	0.38	6.00	67.6	DNA/Helitron
CgiSat06	622	0.33	5.13	67.4	DNA/Helitron
CgiSat07	2091	0.21	3.24	67.0	DNA/Helitron
CgiSat08	166	0.19	2.96	67.5	DNA/Helitron
CgiSat09	166	0.15	2.33	63.3	DNA/Helitron
CgiSat10	433	0.14	2.26	63.0	DNA/Helitron
CgiSat11	892	0.14	2.21	64.6	LTR/BEL
CgiSat12	446	0.11	1.78	64.8	-
CgiSat13	2414	0.11	1.74	67.4	DNA/Kolobok
CgiSat14	1144	0.11	1.74	68.9	DNA/Helitron
CgiSat15	177	0.08	1.32	68.9	-
CgiSat16	2414	0.07	1.12	66.8	DNA/Kolobok
CgiSat17	134	0.06	0.92	76.1	DNA/Helitron
CgiSat18	731	0.05	0.86	63.9	Interspersed Repeat
CgiSat19	212	0.04	0.60	72.6	-
CgiSat20	662	0.05	0.81	65.4	-
CgiSat21	441	0.05	0.77	69.2	DNA/Helitron
CgiSat22	743	0.04	0.66	53.3	LTR/Gypsy
CgiSat23	1295	0.04	0.60	64.9	DNA/MuDR
CgiSat24	66	0.04	0.58	57.6	-
CgiSat25	88	0.03	0.49	61.4	NonLTR/R1
CgiSat26	1894	0.02	0.30	60.4	LTR/Gypsy
CgiSat27	99	0.02	0.26	60.6	-
CgiSat28	182	0.02	0.25	44.0	DNA/MuDR
CgiSat29	344	0.01	0.23	52.9	DNA/DNA4-44
CgiSat30	48	0.01	0.22	68.7	-
CgiSat31	393	0.01	0.18	52.2	IntegratedVirus/DNAV
CgiSat32	536	0.01	0.17	66.6	DNA/Mariner
CgiSat33	105	0.05	0.80	46.7	-
CgiSat34	1423	0.04	0.57	67.3	DNA/DNA3-8
CgiSat35	690	0.02	0.32	68.8	DNA/IS3EU
CgiSat36	45	0.01	0.16	60.0	-
CgiSat37	177	0.34	5.37	56.5	DNA/Helitron
CgiSat38	3287	0.10	1.58	68.0	DNA/DNA2-7
CgiSat39	179	0.05	0.82	52.0	-
CgiSat40	673	0.04	0.65	62.9	DNA/Crypton
CgiSat41	1736	0.02	0.24	66.5	DNA/Ginger1
CgiSat42	930	0.03	0.52	66.9	DNA/DNA4-31
CgiSat43	21	0.01	0.16	66.7	-
CgiSat44	748	0.01	0.16	68.6	LTR/DIRS
CgiSat45	1576	0.08	1.31	64.1	DNA/DNA4-2
CgiSat46	149	0.03	0.41	54.4	-
CgiSat47	129	0.02	0.30	69.8	DNA/IS3EU
CgiSat48	2056	0.40	6.32	64.8	DNA/Helitron
CgiSat49	1084	0.04	0.57	69.4	DNA/DNA2-12
CgiSat50	2078	0.02	0.33	64.1	-
CgiSat51	396	0.01	0.19	63.1	LTR/Gypsy
CgiSat52	1656	0.06	0.92	61.5	DNA/Polinton

**Table 2:** Distribution of the 52 satDNAs constituting the satellitome of *C. gigas* on the chromosomes of two genome assembly datasets.

	Assembly Accession: GCA_902806645.1															Assembly Accession: GCA_011032805.1												
satDNA	LINKAGE GROUP										No. of Chromosomes Occupied	No. of Monomers on Chromosomes	No. of Monomers on Unplaced Scaffolds	Average No. of Monomers Per Chromosome	satDNA	CHROMOSOME										No. of Chromosomes Occupied	No. of Monomers on Chromosomes	Average No. of Monomers Per Chromosome
	1	2	3	4	5	6	7	8	9	10						1	2	3	4	5	6	7	8	9	10			
CgiSat01	+	+	+	+	+	+	+	+	+	+	10	28,120	3946	2812	CgiSat01	+	+	+	+	+	+	+	+	+	+	10	28,486	2849
CgiSat02	+	+	+	+	+	+	+	+	+	+	10	6499	1249	650	CgiSat02	+	+	+	+	+	+	+	+	+	+	10	5563	556
CgiSat03	+	+	+	+	+	+	+	+	+	+	10	2502	411	250	CgiSat03	+	+	+	+	+	+	+	+	+	+	10	2116	212
CgiSat04	+	+	+	+	+	+	+	+	+	+	10	223	32	22	CgiSat04	+	+	+	+	+	+	+	+	+	+	10	198	20
CgiSat05	+	+	+	+	+	+	+	+	+	+	10	1811	312	181	CgiSat05	+	+	+	+	+	+	+	+	+	+	10	1553	155
CgiSat06	+	+	+	+	+	+	+	+	+	+	10	1628	202	163	CgiSat06	+	+	+	+	+	+	+	+	+	+	10	1574	157
CgiSat07	+	+	+	−	+	+	+	+	+	+	9	55	11	6	CgiSat07	+	+	+	+	+	+	+	+	+	+	10	48	5
CgiSat08	+	+	+	+	+	+	+	+	+	+	10	5171	1030	517	CgiSat08	+	+	+	+	+	+	+	+	+	+	10	4949	495
CgiSat09	+	+	+	+	+	+	+	+	+	+	10	4411	406	441	CgiSat09	+	+	+	+	+	+	+	+	+	+	10	4609	461
CgiSat10	+	+	+	+	+	+	+	+	+	+	10	1082	328	108	CgiSat10	+	+	+	+	+	+	+	+	+	+	10	1463	146
CgiSat11	−	+	−	−	+	−	+	−	−	−	3	83	50	28	CgiSat11	+	−	+	+	+	−	+	−	−	−	5	150	30
CgiSat12	−	+	+	−	+	+	−	+	+	−	6	244	21	41	CgiSat12	+	+	+	+	+	−	+	+	−	+	8	672	84
CgiSat13	−	+	+	+	+	+	+	+	+	+	9	39	2	4	CgiSat13	+	+	−	+	+	−	+	+	+	−	7	39	6
CgiSat14	+	+	+	+	+	+	+	+	+	+	10	441	119	44	CgiSat14	+	+	+	+	+	+	+	+	+	+	10	257	26
CgiSat15	+	+	+	+	+	+	+	+	+	+	10	1768	163	177	CgiSat15	+	+	+	+	+	+	+	+	+	+	10	1897	190
CgiSat16	−	+	+	+	+	+	+	+	+	+	9	66	1	7	CgiSat16	+	+	+	+	−	+	−	−	+	−	6	34	6
CgiSat17	+	+	+	+	+	+	+	+	+	+	10	1619	309	162	CgiSat17	+	+	+	+	+	+	+	+	+	+	10	1153	115
CgiSat18	+	+	+	+	+	+	+	+	+	+	10	363	25	36	CgiSat18	+	+	+	+	+	+	+	+	+	+	10	372	37
CgiSat19	+	+	+	+	+	+	+	+	+	+	10	947	352	95	CgiSat19	+	+	+	+	+	+	+	+	+	+	10	1304	130
CgiSat20	−	+	+	−	+	+	+	+	+	−	7	148	54	21	CgiSat20	+	−	−	−	+	+	+	+	−	−	5	245	49
CgiSat21	+	+	+	+	+	+	+	+	+	+	10	542	109	54	CgiSat21	+	+	+	+	+	+	+	+	+	+	10	505	51
CgiSat22	−	−	−	+	−	−	−	−	−	−	1	45	0	45	CgiSat22	−	−	−	−	−	−	−	+	−	−	1	31	31
CgiSat23	+	+	+	+	+	+	+	+	+	−	9	78	14	9	CgiSat23	+	+	+	+	+	+	+	+	+	+	10	148	15
CgiSat24	+	+	+	+	+	+	+	+	+	+	10	2740	128	274	CgiSat24	+	+	+	+	+	+	+	+	+	+	10	2829	283
CgiSat25	+	+	+	+	+	+	+	+	+	+	10	2468	67	247	CgiSat25	+	+	+	+	+	+	+	+	+	+	10	2983	298
CgiSat26	−	−	−	−	−	−	−	+	−	−	1	13	0	13	CgiSat26	−	−	−	+	−	−	−	−	−	−	1	15	15
CgiSat27	−	+	+	+	+	−	+	+	+	−	7	2030	57	290	CgiSat27	+	−	+	+	+	−	+	+	−	+	7	1554	222
CgiSat28	+	+	+	+	+	+	+	+	+	+	10	487	167	49	CgiSat28	+	+	+	+	+	+	+	+	+	+	10	548	55
CgiSat29	+	+	+	+	+	+	+	+	+	+	10	277	19	28	CgiSat29	+	+	+	+	+	+	+	+	+	+	10	267	27
CgiSat30	−	−	−	+	−	+	−	−	−	−	2	2	1	1	CgiSat30	−	−	−	−	+	−	−	+	−	−	2	4	2
CgiSat31	+	−	−	−	−	−	−	−	−	−	1	52	0	52	CgiSat31	−	−	−	−	−	−	+	−	−	−	1	61	61
CgiSat32	−	+	+	−	+	+	−	−	−	−	4	147	0	37	CgiSat32	+	−	−	−	−	−	+	−	−	−	2	75	38
CgiSat33	−	+	+	+	+	+	+	+	+	+	9	3191	254	355	CgiSat33	−	+	−	+	+	+	+	+	+	+	8	2789	349
CgiSat34	−	+	−	−	−	−	−	+	+	+	4	12	2	3	CgiSat34	+	+	−	+	+	+	+	+	+	+	9	43	5
CgiSat35	−	+	−	−	+	+	−	−	−	+	4	6	0	2	CgiSat35	−	−	+	−	−	−	+	−	−	−	2	4	2
CgiSat36	+	−	−	−	−	−	−	−	−	−	1	2	57	2	CgiSat36	−	−	−	−	+	+	+	−	−	+	4	96	24
CgiSat37	+	+	+	+	+	+	+	+	+	+	10	6367	516	637	CgiSat37	+	+	+	+	+	+	+	+	+	+	10	6242	624
CgiSat38	+	+	−	+	−	+	−	+	−	−	5	6	0	1	CgiSat38	+	−	+	−	−	+	−	+	+	−	5	10	2
CgiSat39	+	+	+	+	+	+	+	+	+	+	10	1617	64	162	CgiSat39	+	+	+	+	+	+	+	+	+	+	10	1235	124
CgiSat40	+	+	+	+	+	+	+	+	+	+	10	402	31	40	CgiSat40	+	+	+	+	+	+	+	+	+	+	10	402	40
CgiSat41	−	+	−	−	−	−	−	−	−	−	1	11	0	11	CgiSat41	+	−	−	−	−	−	−	−	−	−	1	7	7
CgiSat42	+	+	+	−	+	+	+	+	−	−	7	124	31	18	CgiSat42	+	−	+	+	+	−	+	−	−	−	5	104	21
CgiSat43	+	+	+	+	+	+	+	+	+	+	10	3145	380	315	CgiSat43	+	+	+	+	+	+	+	+	+	+	10	3416	342
CgiSat44	+	+	+	+	+	+	+	+	+	+	10	54	2	5	CgiSat44	+	+	+	+	+	+	+	+	+	+	10	80	8
CgiSat45	−	+	−	−	−	−	−	−	−	−	1	1	11	1	CgiSat45	+	−	−	−	−	−	−	−	−	−	1	4	4
CgiSat46	+	−	−	−	+	−	−	−	−	+	3	25	0	8	CgiSat46	−	+	−	+	+	+	−	+	+	−	6	359	60
CgiSat47	+	+	+	−	+	+	−	+	+	−	7	251	79	36	CgiSat47	+	−	+	+	+	+	+	+	+	+	9	340	38
CgiSat48	−	−	+	+	+	+	+	+	−	+	7	20	4	3	CgiSat48	+	+	+	+	−	+	−	−	−	+	6	10	2
CgiSat49	+	+	+	+	+	+	+	+	+	+	10	77	4	8	CgiSat49	+	+	+	+	+	+	+	+	+	+	10	79	8
CgiSat50	−	−	−	−	−	−	−	−	−	−	0	0	0	0	CgiSat50	−	−	−	−	−	−	−	−	−	−	0	0	0
CgiSat51	−	−	−	+	−	+	−	−	−	−	2	122	0	61	CgiSat51	−	+	−	−	−	−	−	+	−	−	2	87	44
CgiSat52	+	−	−	−	−	−	−	−	−	−	1	17	0	17	CgiSat52	−	−	−	−	−	−	+	−	−	−	1	6	6

For each satDNA, presence (+) or absence (−) and total number of monomers present on chromosomes and unplaced scaffolds are indicated.

## Data Availability

The data presented in this study are available in NCBI GenBank under the BioSample accession number: SAMN15184427, BioProject: PRJNA638244. All scripts used are available at: https://bitbucket.org/MonikaTC/tunjic-cvitanic-et-al.-2021/src/master/ (added on 1 May 2021).

## Supplementary Materials

The following are available online at https://www.mdpi.com/article/10.3390/ijms22136798/s1.
