# Supplementary material for: Satellitome Analysis of the Pacific Oyster Crassostrea gigas Reveals New Pattern of Satellite DNA Organization, Highly Scattered across the Genome
Source: Int J Mol Sci. 2021 Jun 24;22(13):6798. doi: 10.3390/ijms22136798 (PMC8268682; doi:10.3390/ijms22136798)
Supplement: Supplementary file 1 [file ijms-22-06798-s001.zip › Supplementary Materials.pdf]

Satellitome Analysis of the Pacific oyster *Crassostrea gigas* reveals new pattern of satellite DNA organization, highly scattered across the genome

Monika Tunjić-Cvitanić, Juan J. Pasantes, Daniel García-Souto, Tonči Cvitanić, Miroslav Plohl, Eva Šatović-Vukšić

Supplementary Materials

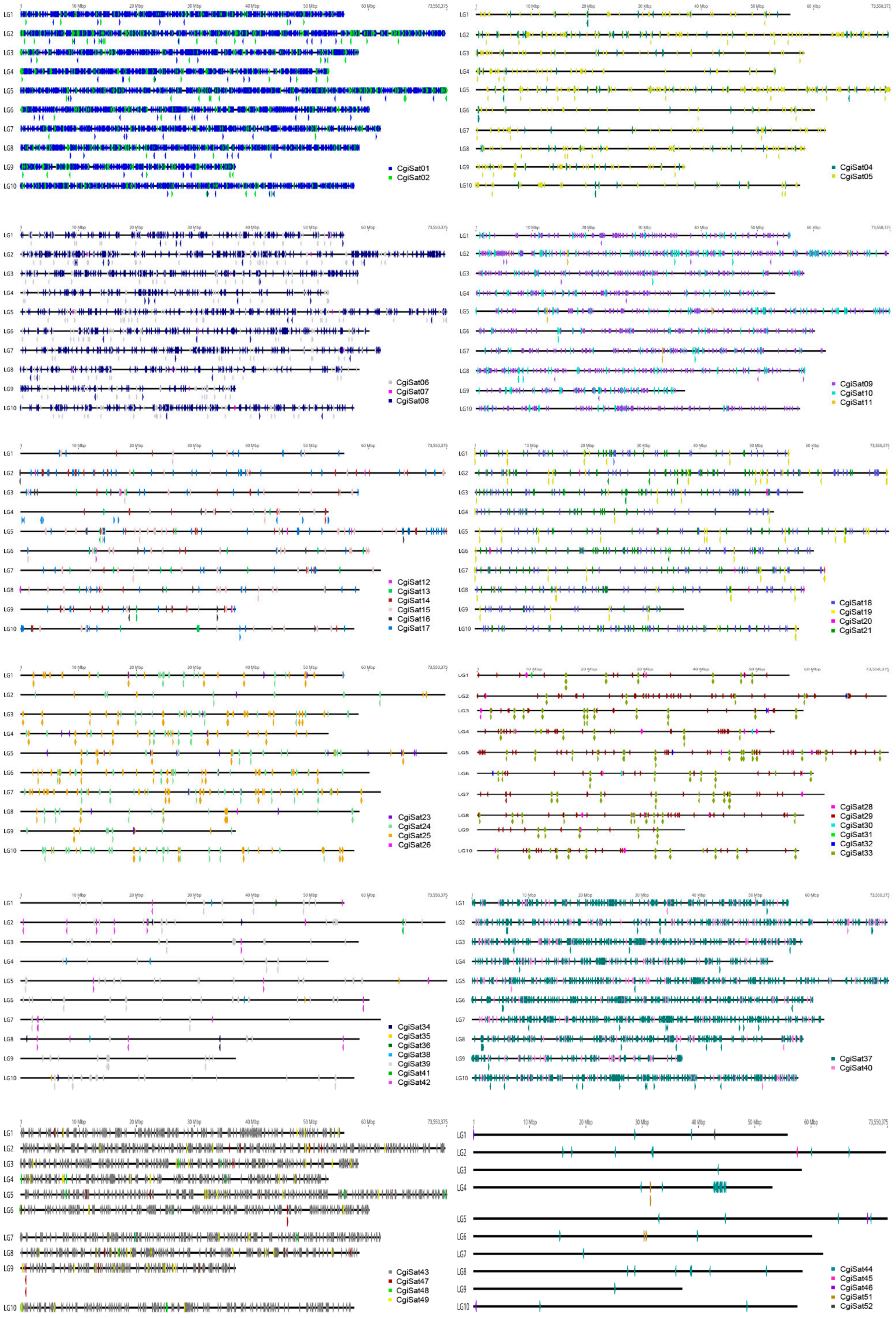
1
10 Mbp
20 Mbp
30 Mbp
40 Mbp
50 Mbp
60 Mbp
73,550,375
LG1
LG2
LG3
LG4
LG5
LG6
LG7
LG8
LG9
LG10
CgiSat01
CgiSat02
CgiSat04
CgiSat05
CgiSat06
CgiSat07
CgiSat08
CgiSat09
CgiSat10
CgiSat11
CgiSat12
CgiSat13
CgiSat14
CgiSat15
CgiSat16
CgiSat17
CgiSat18
CgiSat19
CgiSat20
CgiSat21
CgiSat23
CgiSat24
CgiSat25
CgiSat26
CgiSat28
CgiSat29
CgiSat30
CgiSat31
CgiSat32
CgiSat33
CgiSat34
CgiSat35
CgiSat36
CgiSat38
CgiSat39
CgiSat41
CgiSat42
CgiSat37
CgiSat40
CgiSat43
CgiSat47
CgiSat48
CgiSat49
CgiSat44
CgiSat45
CgiSat46
CgiSat51
CgiSat52

Figure S1. *In silico* localization of all RepeatExplorer2-detected satDNAs of *C. gigas* (except CgiSat03, CgiSat27, and CgiSat22 presented in Figure 2), annotated on the chromosomes of the *C. gigas* representative genome assembly GCA_902806645.1. Some satDNA monomers are automatically placed below the black bar representing the chromosome due to the inability of the software to mark on the chromosome all the repeats that are in close proximity to each other.

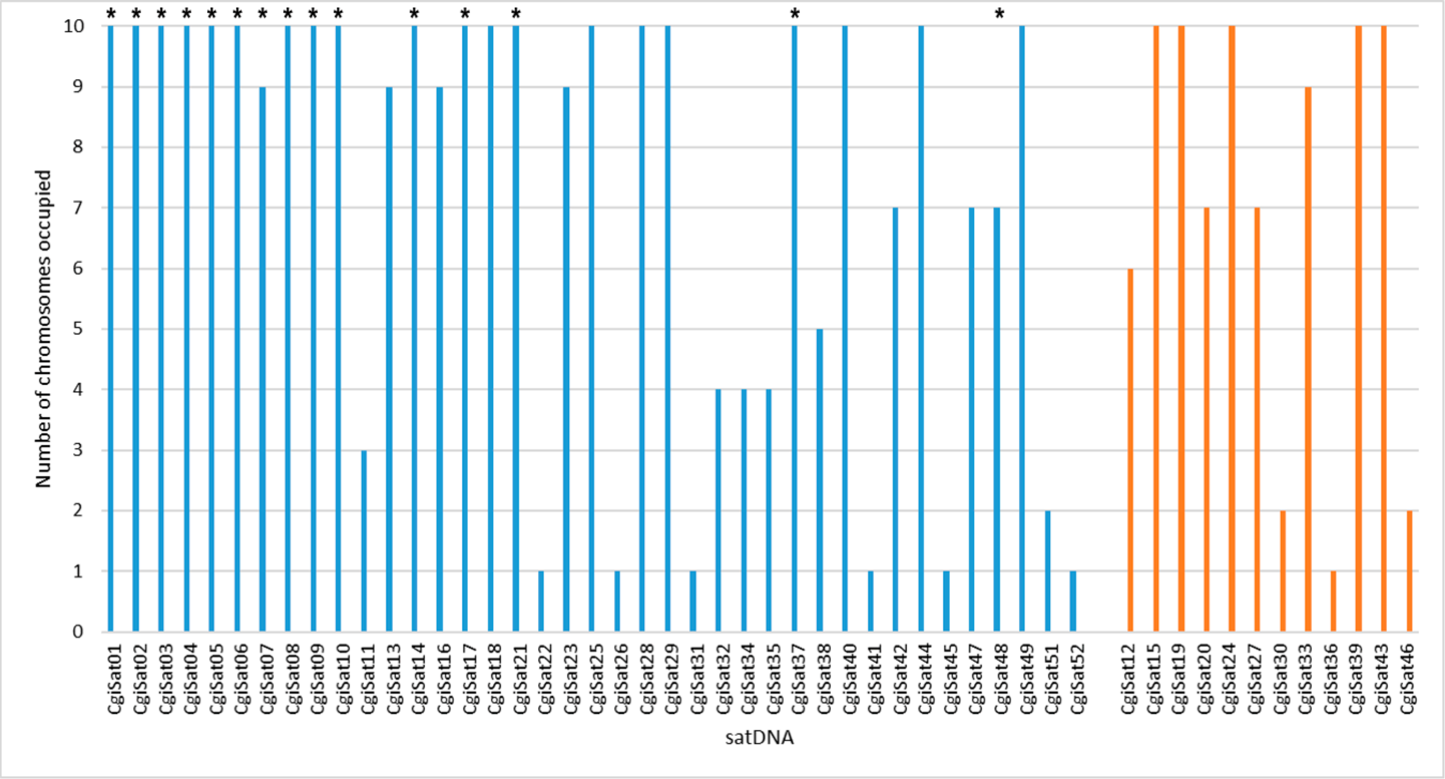

Figure S2. Chromosomal distribution of the 52 satDNAs composing the satellitome of *C. gigas*. Number of chromosomes of the representative genome assembly GCA_902806645.1 in which satDNAs related (blue) and unrelated (orange) to mobile elements appear. Asterisks denote satDNAs showing similarity to Helitron mobile elements.

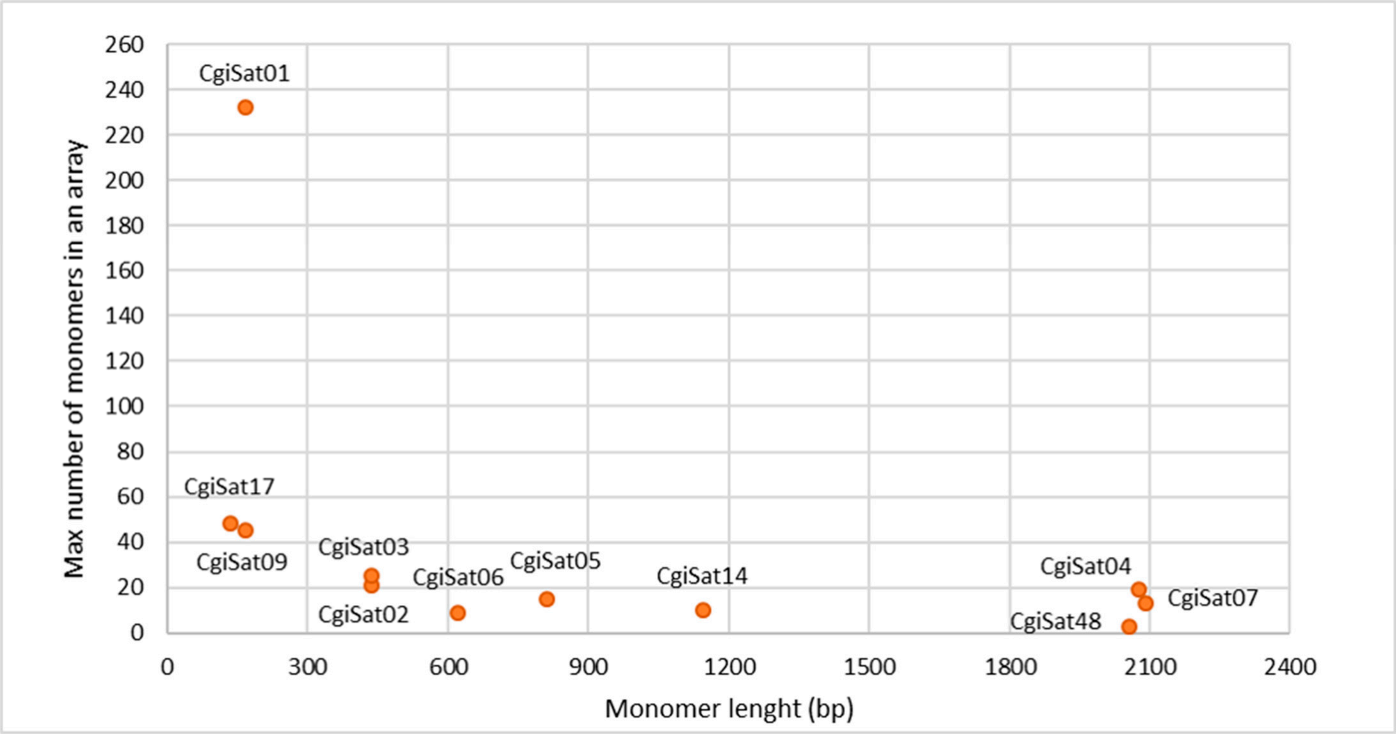

Figure S3. Relation between the maximum number of monomers detected in an array and the monomer size for eleven *C. gigas* satDNAs.

Table S1. Outputs of the four rounds of RepeatExplorer2 clustering performed on subsampled sets of reads.

	Clustering 1		Clustering 2		Clustering 3		Clustering 4	
satDNA	1 779 522 reads, 0.35 x genome coverage	% of the genome	1 000 000 reads, 0.2 x genome coverage	% of the genome	2 000 000 reads, 0.4 x genome coverage	% of the genome	1 000 000 reads, 0.2 x genome coverage	% of the genome
**CgiSat01a**	Cl1	0.76	Cl1	0.79	Cl1	0.79	Cl2	0.52
**CgiSat01b**	Cl2	0.57	Cl2	0.57	Cl2	0.58	Cl1	0.57
**CgiSat02**	Cl4	0.40	Cl3	0.46	Cl3	0.48	Cl4	0.41
**CgiSat03**	Cl5	0.38	Cl4	0.39	Cl7	0.39	Cl9	0.36
**CgiSat04**	Cl8	0.36	Cl9	0.32	Cl10	0.33	-	-
**CgiSat05**	Cl9	0.35	Cl5	0.38	Cl6	0.39	Cl7	0.40
**CgiSat06**	Cl11	0.33	Cl8	0.32	Cl11	0.33	Cl11	0.32
**CgiSat07**	Cl28	0.20	Cl22	0.21	Cl26	0.20	Cl23	0.21
**CgiSat08**	Cl29	0.19	Cl27	0.19	Cl29	0.19	Cl30	0.18
**CgiSat09**	Cl40	0.16	Cl48	0.13	Cl40	0.16	Cl41	0.14
**CgiSat10**	Cl46	0.14	-	-	Cl44	0.15	Cl40	0.14
**CgiSat11**	Cl48	0.14	Cl43	0.14	Cl48	0.14	Cl39	0.14
**CgiSat12**	Cl59	0.12	Cl61	0.10	Cl63	0.12	Cl56	0.11
**CgiSat13**	Cl60	0.11	Cl59	0.11	-	-	-	-
**CgiSat14**	Cl61	0.11	Cl58	0.11	Cl70	0.11	-	-
**CgiSat15**	Cl86	0.09	Cl80	0.08	Cl85	0.09	Cl77	0.08
**CgiSat16**	Cl102	0.07	Cl91	0.07	Cl103	0.07	Cl89	0.07
**CgiSat17**	Cl119	0.06	Cl122	0.06	Cl128	0.06	-	-
**CgiSat18**	Cl129	0.06	Cl121	0.06	-	-	Cl130	0.05
**CgiSat19**	Cl139	0.05	Cl180	0.04	Cl199	0.04	Cl206	0.03
**CgiSat20**	Cl145	0.05	Cl138	0.05	Cl152	0.05	Cl125	0.05
**CgiSat21**	Cl152	0.05	Cl137	0.05	Cl148	0.05	Cl142	0.05
**CgiSat22**	Cl175	0.04	Cl159	0.04	Cl173	0.04	Cl152	0.04
**CgiSat23**	Cl182	0.04	Cl175	0.04	-	-	-	-
**CgiSat24**	Cl199	0.03	Cl171	0.04	Cl194	0.04	Cl173	0.04
**CgiSat25**	Cl205	0.03	-	-	Cl211	0.03	Cl204	0.03

**CgiSat26**	Cl269	0.02	Cl247	0.02	Cl293	0.02	Cl246	0.02
**CgiSat27**	Cl296	0.02	Cl266	0.02	Cl316	0.02	Cl262	0.02
**CgiSat28**	Cl298	0.02	Cl264	0.02	Cl301	0.02	Cl282	0.02
**CgiSat29**	Cl308	0.02	-	-	-	-	Cl300	0.01
**CgiSat30**	Cl326	0.01	Cl295	0.01	Cl332	0.01	Cl290	0.02
**CgiSat31**	Cl343	0.01	-	-	Cl362	0.01	Cl344	0.01
**CgiSat32**	Cl373	0.01	-	-	Cl365	0.01	-	-
**CgiSat33**	-	-	Cl139	0.05	Cl147	0.05	-	-
**CgiSat34**	-	-	Cl186	0.03	Cl187	0.04	-	-
**CgiSat35**	-	-	Cl234	0.02	Cl274	0.02	-	-
**CgiSat36**	-	-	Cl341	0.01	Cl390	0.01	Cl341	0.01
**CgiSat37**	Cl10	0.34	-	-	-	-	-	-
**CgiSat38**	Cl70	0.10	-	-	-	-	-	-
**CgiSat39**	Cl142	0.05	-	-	-	-	-	-
**CgiSat40**	Cl178	0.04	-	-	-	-	-	-
**CgiSat41**	Cl312	0.02	-	-	-	-	-	-
**CgiSat42**	-	-	Cl187	0.03	-	-	-	-
**CgiSat43**	-	-	Cl342	0.01	-	-	-	-
**CgiSat44**	-	-	Cl344	0.01	-	-	-	-
**CgiSat45**	-	-	-	-	Cl93	0.08	-	-
**CgiSat46**	-	-	-	-	Cl241	0.03	-	-
**CgiSat47**	-	-	-	-	Cl282	0.02	-	-
**CgiSat48**	-	-	-	-	-	-	Cl5	0.40
**CgiSat49**	-	-	-	-	-	-	Cl172	0.04
**CgiSat50**	-	-	-	-	-	-	Cl234	0.02
**CgiSat51**	-	-	-	-	-	-	Cl319	0.01
**CgiSat52**	-	-	-	-	-	-	Cl111	0.06

Cluster names and genomic abundance are provided for each satDNA cluster. Combining the data from the four analyses yielded a satellitome composed of 52 satDNAs.

Table S2. Helitron elements showing similarity to *C. gigas* satDNAs in the Repbase search.

**satDNA**	**Repbase classification**
CgiSat01	Helitron-N2_Cgi
CgiSat02	Helitron-N40_CGi
CgiSat03	Helitron-N31_CGi
CgiSat04	Helitron-N3_CGi
CgiSat05	Helitron-N4_CGi
CgiSat06	Helitron-N32_CGi
CgiSat07	Helitron-N3_CGi
CgiSat08	Helitron-N25_CGi
CgiSat09	Helitron-N2C_Cgi
CgiSat10	Helitron-N62B_CGi
CgiSat14	Helitron-N28_CGi
CgiSat17	Helitron-N35_CGi
CgiSat21	Helitron-1_DEu
CgiSat37	Helitron-N12_CGi
CgiSat48	Helitron-N29_CGi

Table S3. Nucleotide sequences of the five Helitron conserved boxes.

**Box**	**Nucleotide sequence**
Box 1	TAGCTCACCTGAGCCGAAGGCTCAAGTGAGCTTTTCTGATCACA
Box 2	TGTTGCTCAGGTGAGCGATGTGGCCCATGGGCCTCTTGT
Box 3	AAACTTTTCACATTTTCAACTTCTTCTCAAGAACCACTGGGCCAATTTCAACC<br>AAATTTGGCACAAAGCATCCTTAGGBDAAGGGGATTHAAATTTGTTAAAATD<br>AAGGGCCACGCCCTTTTBAAAAGGGAGATAATTGVGAATTADTGAAAATTT
Box 4	TAGGGTCTTCCGTTTTCCAACGGAAGACCCTATTGTTATTGTTCTGTTTCTTT
Box 5	ACGGAAGACCCACTCGTTGCTCGCAACGAGATCGTGTCTAGT

Table S4. Positions of the satDNA sequences on the chromosomes or scaffolds, number of monomers in each extraction, and presence (1) or absence (0) of Helitron boxes in the 2000 bp flanking the satDNA sequence for eleven of satDNAs.

The table is provided as a separate **Table S4.xsls** document containing 11 sheets.

Table S5. Primers and parameters used in PCR reactions for probe labelling.

Primers used	Initial denaturation	Amplification (30 cycles)	Final extension
CgiSat02_F: TAGGGGTTTTTAGGGGCCAA<br>CgiSat02_R: TAGGGGCCGATCCCTTATCTC	94 °C/5 m	94 °C/30 s<br>56 °C/30 s<br>72 °C/30 s	72 °C/ 7 m
CgiSat03_F: CCTCTAGATCCTTAATGGGGACA<br>CgiSat03_R: GCTGGCCCCTAAAATATTCAATCA		94 °C/30 s<br>58 °C/30 s<br>72 °C/30 s	
CgiSat04_F: TGCAGCCATTTTGCTGTCTG<br>CgiSat04_R: CAGCTCGCCCTACATGTGAT		94 °C/30 s<br>55 °C/30 s<br>72 °C/30 s	
CgiSat05_F: GGAAGTAAAGCTGGAAGTTCCTGTA<br>CgiSat05_R: TGTCACTTCCGGTCGAAACC		94 °C/30 s<br>58 °C/30 s<br>72 °C/30 s	
CgiSat09_F: GTTAAACTTTGAACCCCGCCT<br>CgiSat09_R: CTCAAGAACCACTGCACCAGA		94 °C/30 s<br>58 °C/30 s<br>72 °C/30 s	
CgiSat17_F: TCGTGTTTAAATGACCTCAGAC<br>CgiSat17_R: GCAGAATTGTAAGCGCAACTT		94 °C/30 s<br>62 °C/30 s<br>72 °C/30 s	
CgiSat28_F: GTTTAGATCCCTACATAACAG<br>CgiSat28_R: TAGGACTTGTGCTCATTAGG		94 °C/30 s<br>60 °C/30 s<br>72 °C/30 s	
CgiSat37_F: AGCATCCGAAACCTATGGCTC<br>CgiSat37_R: TTGCAGGTGCCCTCTGATGA		94 °C/30 s<br>56 °C/30 s<br>72 °C/30 s	
CgiSat46_F: TTGTACAGAGGTAGGTCAC<br>CgiSat46_R: TTAAACTTCCGGGTTGGG		94 °C/30 s<br>47 °C/30 s<br>72 °C/30 s	
5S rDNA_F: GAAGTTAAGCAACGTAGAG<br>5S rDNA_R: TCAATCTTTCTATCTGTG		94 °C/30 s<br>43 °C/30 s<br>72 °C/30 s	

Data S1. Fasta files of the consensus sequences of the 52 satDNAs constituting the satellitome of *C. gigas.*

The file is provided as a separate document.
